# Anti-ICAM-1 antibody-modified nanostructured lipid carriers: a pulmonary vascular endothelium-targeted device for acute lung injury therapy

**DOI:** 10.1186/s12951-018-0431-5

**Published:** 2018-12-29

**Authors:** Shujuan Li, Li Chen, Guokang Wang, Lexing Xu, Shanshan Hou, Ziwei Chen, Xiaoling Xu, Xiaojuan Wang, Fuhe Liu, Yong-Zhong Du

**Affiliations:** 1 Department of Pharmacy, Zhejiang Pharmaceutical College, Ningbo, 315100 Zhejiang China; 2 Institute of Pharmaceutics, College of Pharmaceutical Sciences, Zhejiang University, 866 Yuhangtang Road, Hangzhou, 310058 People’s Republic of China; 3 Department of Pharmacy, The First Affiliated Hospital, College of Medicine, Zhejiang University, Hangzhou, 310003 China

**Keywords:** Acute lung injury, Pulmonary vascular endothelium-targeting, ICAM-1, Dexamethasone delivery, Surface charge

## Abstract

**Background:**

Acute lung injury (ALI) is a life-threatening clinical syndrome without effective treatment. Targeting delivery of glucocorticoid to lung shows potential efficacy for ALI based on their anti-inflammatory and anti-fibrotic properties, breaking through their clinical application limitation due to systemic side effects. This work was aimed to establish lung-targeted dexamethasone (DEX) loaded nanostructured lipid carriers (NLCs) with opposite surface charge and investigate their therapeutic effects on lipopolysaccharide (LPS)-induced ALI mice.

**Results:**

The diameter of anionic anti-intercellular adhesion molecule 1 (anti-ICAM-1) antibody-conjugated DEX-loaded NLCs (ICAM/DEX/NLCs) and the cationic ones with octadecylamine (ODA) modification (ICAM/DEX/ODA-NLCs) was about 249.9 and 235.9 nm. The zeta potential of ICAM/DEX/NLCs and ICAM/DEX/ODA-NLCs was about − 30.3 and 37.4 mV, respectively. Relative to the non-targeted control and ICAM/DEX/ODA-NLCs, ICAM/DEX/NLCs exhibited higher in vitro cellular uptake in LPS-activated human vascular endothelial cell line EAhy926 after CAM-mediated endocytosis, and stronger in vivo pulmonary distribution in the ALI model mice. In vivo i.v. administration of ICAM/DEX/NLCs significantly attenuated pulmonary inflammatory cells infiltration, and the production of pro-inflammatory cytokine TNF-α and IL-6 in ALI mice. H&E stain also revealed positive histological improvements by ICAM/DEX/NLCs.

**Conclusions:**

ICAM/DEX/NLCs may represent a potential pulmonary endothelium targeted device, which facilitate translation of DEX into clinical ALI treatment.

**Electronic supplementary material:**

The online version of this article (10.1186/s12951-018-0431-5) contains supplementary material, which is available to authorized users.

## Background

Acute lung injury (ALI) is an inflammatory disease process of the lungs resulting from sepsis, pneumonia or other insults, which could further develop as acute respiratory distress syndrome (ALI) even pulmonary fibrosis [1]. Numerous research and therapeutic trials have been developed to improve ALI, including administration of relevant growth factors, stem cell therapy and interventions to block coagulation/complement cascade, etc. [2]. Other supportive care such as prone positioning, recruitment maneuvers and positive end-expiratory pressure (PEEP) are also employed in clinical treatment [3]. However, no proven therapeutic modality is reported as an effective treatment for ALI in clinic, the mortality rate of ALI/ARDS still remains high.

Great interest remains in the use of glucocorticoid for the salvage of ALI patients, given their potential anti-inflammatory and anti-fibrotic characteristics [4]. The glucocorticoid showed potentials of inducing anti-inflammatory cytokines expression including IL-10 and inhibiting pro-inflammatory cytokines expression including TNF-α, IL-6 and IL-8, which was of great significance to attenuate the excessive pulmonary inflammation of ALI [5]. Although there exists controversy on the glucocorticoid treatment for ALI, the positive effects of glucocorticoid on clinical ARDS improvements have still been reported widely, such as the reduction of systemic inflammation and the ICU length of stay, etc. [6–9]. Noteworthily, low-dose glucocorticoid administered (e.g. up to 2 mg/kg/day of methylprednisolone) in early ALI/ARDS was suggested to reduce the mortality in patients, while high-dose glucocorticoid was widely not recommend due to the disadvantages such as the increasing risk of infection [6, 8]. This highlighted the significance of delivering sufficient therapeutic cargoes to lung with a relative low-dose glucocorticoid regimen for efficacy and safety. Besides, prolonged glucocorticoid treatment was reported to provide improved ALI/ARDS outcomes [10], further suggesting a well-tolerated treatment was required. Therefore, exploring a method to efficiently deliver glucocorticoid to inflammatory lung and minimize the potential disadvantages (infection, hyperglycaemia and gastrointestinal bleeding, etc.) is of great significance in clinic.

Targeted drug-delivery systems (TDDS) potentially deliver therapeutic cargos to diseased tissues or specific organelles, increasing therapeutic efficacy and decreasing adverse effects [11, 12]. The major nanocarriers such as liposomes, dendrimers, polymeric nanoparticles, micelles, lipid nanoparticles and so on were studied widely for drug delivery due to their distinguishing advantages (tunable size, modifiable surface, controlled drug release, etc.) [13, 14]. For example, mitochondrial-targeted liposomes loaded with paclitaxel were prepared to enhance cytotoxicity toward cancer cells [12]. Folic acid targeted dendrimers with covalently conjugated methotrexate were developed to specifically kill folate receptor-expressing cells [15]. Nanostructured lipid carriers (NLCs), the second generation of lipid nanoparticles with numerous superiorities including excellent biocompatibility, feasibility of large-scale production and so on, have attracted great interests as potential TDDS [16]. Targeted delivery of drugs by TDDS to lesion could be realized though their passive targeting and active targeting characteristics. Primarily, the carriers can passively accumulate in the interested sites through passive targeting by modulating physical parameters including particle surface charge, diameter, surface modification and so on, which were closely associated with the DDS in vivo fate. For example, it has been reported that anionic nanoparticles have prolonged in vivo circulation time relative to the cationic ones, which potentially undergo rapid clearance from the blood due to the interaction with serum proteins [17]. However, one has to recognize that drugs delivery to pathological site is generally limited via passive targeting, whereas it could be further enhanced via active targeting.

A suitable targeted site plays a vital role for TDDS anchoring and accumulation in the pathological foci. ICAM, a transmembrane glycoprotein preferentially expressed on endothelial cells (ECs), could be dramatically over-expressed under inflammation states. Binding of the circulating DDS with ICAM-1 epitope expressed on endothelium potentially enhances pulmonary accumulation of the particles due to (i) the extended pulmonary luminal surface area (~ 25% of the whole endothelium surface in the body), (ii) the collection of entire cardiac output of venous blood from right ventricle before other organs sharing the arterial output, (iii) the over-expressed ICAM-1 on pulmonary endothelium under ALI relative to the modest expression on normal endothelium of the body, and (iv) low blood flow rate in pulmonary microcirculation [18]. Coupling nanoparticle’s surface with affinity ligands of ICAM-1 epitope such as anti-ICAM-1 antibody potentially realizes the lung-targeted delivery of drugs and the reduction of non-target effects on normal organs [19].

In this study, dexamethasone (DEX), the long-acting and high-potency glucocorticoid, was used as the model drug. The anionic and cationic anti-ICAM-1 antibody-conjugated dexamethasone loaded NLCs were developed to explore the effects on LPS-induced ALI mice. The physicochemical properties, cytotoxicity, cellular uptake, cellular transport pathway and bio-distribution of the NLCs were evaluated successively. The in vivo pharmacodynamics after i.v. administration of the NLCs in LPS-induced ALI mice was assessed as well.

## Methods

### Materials

Dexamethasone was obtained from Aladdin Bio-chem Technology Co. Limited (Shanghai, China). Medium-chain triglycerides (MCT) were gifted from Gattefosse (France). Monostearin was obtained from Shanghai Chemical Reagent Co., Ltd. (Shanghai, China). Polyethylene glycol monostearate (PEG_2000_-SA, M_W_ = 2000) was from Tokyo Kasei Kogyo Co., Ltd. (Japan). Octadecylamine (ODA) was from Fluka (USA). Lipopolysaccharide (LPS, Escherichia coli O55:B5), 3-(4,5-dimethylthiazol-2-yl)-2,5-diphenyltetrazolium bromide (MTT) and *N*,*N*′-disuccinimidyl carbonate (DSC) were from Sigma-Aldrich (St. Louis, MO). Mouse anti-human ICAM-1 antibody (6.5B5) and rat anti-mouse ICAM-1 antibody (YN1/1.7.4) were from Santa Cruz Biotechnology, Inc. (Santa Cruz, CA). Anti-IgG antibody was from Sangon Biotech (Shanghai, China). Cy3-labeled goat anti-mouse IgG and ACK Lysis Buffer were from Beyotime Biotechnology Co., Ltd. (Shanghai, China). Near-infrared DiR fluorescent probe was from Life Technologies (Carlsbad, CA). Octadecylamine-fluorescein isothiocyanate (ODA-FITC) and amino-terminated polyethylene glycol monostearate (NH_2_-PEG_2000_-SA) were synthesized previously [20]. All other solvents were of analytical or chromatographic grade.

### Preparation of NLCs

The NLCs were prepared via solvent diffusion method [21]. In brief, 4 mg dexamethasone, 56 mg monostearin, 30 mg MCT, 0.5 mg NH_2_-PEG_2000_-SA and 9.5 mg PEG_2000_-SA were dissolved in 1 mL ethanol in water bath at 60 °C. Subsequently, 0.1 mL dissolved solution was removed and rapidly dispersed into 1.9 mL of deionized water (ethanol:water = 1:19, v/v) at 60 °C under 400 rpm stirring (DC-40, Hangzhou Electrical Engineering Instruments, China) for 2 min. The obtained nano-suspensions were placed at room temperature to solidify the melted lipid droplets. Then 25 μL of 0.2 mg/mL water-DSC solution (NH_2_-PEG_2000_-SA:DSC = 1:1, mol/mol) was sufficiently dispersed in the NLC dispersions via 30 s vortex, followed by 3 h reaction at room temperature with slight shaking. After reaction, 10.4 μg anti-ICAM-1 antibody was added in the dispersion followed by another 3 h slight shaking at room temperature. The anti-ICAM-1 antibody-conjugated dexamethasone loaded NLCs (ICAM/DEX/NLCs) with 5 mg/mL concentration were prepared. To prepare the non-targeted control NLCs, anti-IgG antibody was used instead of anti-ICAM-1 antibody to prepare IgG-modified NLCs (IgG/DEX/NLCs), while the other procedures were the same as ICAM/DEX/NLCs preparation.

Meanwhile, 3 mg octadecylamine (ODA) was used instead of 3 mg monostearin to prepare the ODA modified NLCs with anti-ICAM-1 antibody conjugation (ICAM/DEX/ODA-NLCs) and anti-IgG antibody conjugation (IgG/DEX/ODA-NLCs). The other procedures were the same as mentioned above.

### Characterization of NLCs

The hydrodynamic diameters, polydispersity index (PI) and zeta potential of the nanoparticles were detected by dynamic light scattering using a Zetasizer (3000HS, Malvern Instruments Ltd, UK). A transmission electronic microscopy (TEM, JEM-1200EX, JEOL, Japan) was employed to observe the morphologies of the nanoparticles. The samples were negatively stained using 1% (w/v) uranyl acetate for 1 min followed by being photographed via TEM.

### Drug encapsulation efficiency and drug loading

The content of dexamethasone in the nanoparticles was analyzed using high-performance liquid chromatography system (HPLC) after centrifugal-ultrafiltration [21]. The mobile phase was composed of methanol-deionized water (80:20, v/v) with a flow rate of 1 mL/min and the ultraviolet detection wavelength was at 240 nm [22]. The prepared nano-suspensions were added into a centrifugal-ultrafiltration tube (MWCO 3000, Millipore Co., USA) and centrifuged 15 min at 10,000 rpm for three times to saturate the filter membrane. The dexamethasone concentration in ultrafiltrate after the fourth centrifugation was detected. The encapsulation efficiency (EE%) and drug loading (DL%) of dexamethasone in the nanoparticles were calculated using the following equations:

1 $${\text{EE}}\% \, = \, \left( {{\text{M}}_{\text{a}} {-}{\text{ M}}_{\text{s}} } \right)/{\text{M}}_{\text{a}} \times { 1}00\%$$

2 $${\text{DL}}\% \, = \, \left( {{\text{M}}_{\text{a}} {-}{\text{ M}}_{\text{s}} } \right)/\left( {{\text{M}}_{\text{a}} + {\text{ M}}_{\text{c}} - {\text{ M}}_{\text{s}} } \right) \, \times { 1}00\%$$

where M_s_ is the mass of dexamethasone in ultrafiltrate. M_a_ is the mass of dexamethasone added in the system. M_c_ is the mass of lipid materials added in system.

### In vitro drug release

The in vitro dexamethasone release study was carried out using dialysis method [23]. The prepared dexamethasone-loaded NLCs were diluted to 1 mg/mL (carriers concentration) firstly. Afterwards, 1 mL of the diluted nano-suspensions was transferred into a dialysis bag (MW cut off 7 kDa) followed by immersion into 20 mL phosphate-buffered saline (PBS, pH 7.4) for release study using an incubator shaker at 37 °C (60 rpm, HZ-8812S, Scientific and Educational Equipment plant, Tai Cang, China). 1 mL dissolution medium was withdrawn and 1 mL fresh PBS was added simultaneously at scheduled time points. The dexamethasone released from the formulated NLCs in the dissolution medium was analyzed using HPLC.

### Cells culture

EAhy926 cells (EAs), the human vascular endothelial cell line [24], were obtained from ATCC. The cells were maintained in Dulbecco’s modified eagle medium (DMEM) containing 10% (v/v) fetal bovine serum (FBS) without antibiotic. The humidified cell incubator was set at 37 °C with 5% CO_2_. To establish in vitro cell model of inflammatory pathological endothelium with over-expressed ICAM-1, the EAs were incubated with 400 ng/mL of LPS for 24 h as our previous study reported [20].

### Cytotoxicity study

To evaluate cytotoxicity of the materials and the DEX-loaded nanoparticles, MTT assay was carried out using the blank carriers and the DEX-loaded NLCs on the LPS-activated EAs. The blank carriers (ICAM/NLCs, IgG/NLCs, ICAM/ODA-NLCs and IgG/ODA-NLCs) were prepared using the same lipid ingredients with the drug-loaded NLCs (60 mg monostearin, 30 mg MCT, 0.5 mg NH_2_-PEG_2000_-SA and 9.5 mg PEG_2000_-SA) via solvent diffusion method mentioned above. EAs were transferred to 96-well microtiter plates (5 × 10^3^ cells/well) to grow 24 h followed by 24 h LPS-stimulation. Then the EAs were exposed to blank carriers and the DEX-loaded NLCs of different carriers concentrations (100–600 μg/mL). After 48 h incubation, 20 μL of MTT (5 mg/mL) was added in each well for another 4 h. Afterwards, the supernatant was withdrew and the formazan was dissolved using 150 μL DMSO by shaking (90 rpm, 37 °C, 30 min). A microplate reader (Bio-Rad, model 680, USA) was used to determine the absorption at 570 nm. Cell viability was calculated using the following equation:

$${\text{Cell viability }}\left( \% \right) = \, \left( {{\text{OD}}_{\text{treated cells}}/{\text{OD}}_{\text{control cells}} } \right) \times { 1}00\% .$$

### Cellular internalization study

The octadecylamine-fluorescein isothiocyanate (ODA-FITC) was used as the fluorescence probe to label the NLCs for cellular uptake study [25]. Briefly, 3 mg ODA-FITC was used instead of 3 mg monostearin to prepare ODA-FITC labeled NLCs via solvent diffusion method as mentioned above. The EAs were transferred to 24-well plate (5 × 10^4^ cells/well) to grow 24 h for adherence followed by 24 h LPS-stimulation. Afterwards, the quiescent and activated EAs were incubated with FITC-labeled ICAM/DEX/NLCs, IgG/DEX/NLCs, ICAM/DEX/ODA-NLCs and IgG/DEX/ODA-NLCs for scheduled time. The incubation concentration of the NLCs is 20 μg/mL. At the end of incubation, the supernatant of medium was withdrew and the cells were washed using PBS, followed by fixation with 4% paraformaldehyde (PFA) for 15 min at room temperature. A fluorescence microscope (Leica DM4000 B; Leica, Solms, Germany) was used to observe the cellular fluorescence qualitatively.

The internalization of the nanoparticles was further quantitatively analyzed by a flow cytometer (FC500MCL, Beckman Coulter). The EAs were seeded in 6-well plates (1 × 10^5^ cells/well). The quiescent and LPS-activated EAs were incubated with the formulated NLCs as mentioned above for predetermined time. At the end of incubation, the cells were washed for two times using PBS, followed by being trypsinized and harvested via centrifugation for detection.

### Transport pathway study

The transport pathway study was carried out using ODA-FITC labeled ICAM/DEX/NLCs in EAs. The EAs were transferred to 24-well plate (5 × 10^4^ cells/well) with sterile coverglass to grow 24 h for adherence. Three experimental groups were designed including (i) the activated EAs incubated with ICAM/DEX/NLCs, (ii) the activated EAs blocked with free anti-ICAM-1 antibody (3 μg/mL) for 1 h followed by incubation with ICAM/DEX/NLCs (ICAM-1 blockage study), (iii) the resting EAs incubated with ICAM/DEX/NLCs. The incubation concentration of ICAM/DEX/NLCs is 20 μg/mL. After 2 h incubation, the supernatant of medium was withdrawn and the cells were washed using PBS, followed by fixation with 4% paraformaldehyde (PFA) for 15 min at room temperature. To evaluate the ICAM-1 expression on cells, the fixed resting and activated EAs were incubated with the primary antibody (Mouse anti-human ICAM-1 antibody) overnight at 4 °C followed by incubation with fluorescent secondary antibody (Cy3-labeled goat anti-mouse IgG) for 1 h at room temperature. The cells growing on the coverglass were photographed using a confocal microscopy (OLYMPUS FV1000).

The internalization of the nanoparticles was further quantitatively analyzed by a flow cytometer. The EAs were seeded in 6-well plates (1 × 10^5^ cells/well). The experimental groups were the same as the groups designed in qualitative determination experiment mentioned above. At the end of incubation, the cells were washed by PBS, followed by being trypsinized and harvested via centrifugation for detection.

### Animals test

The male Balb/c mice (7–8 weeks) were purchased from Shanghai Silaike Laboratory Animal Co., Ltd. The animals were housed in a climate-controlled environment, with standard food and water and a 12/12 h light/dark cycle.

### Bio-distribution study

The bio-distribution study was carried out using DiR-labeled NLCs in healthy mice and the ALI model mice. 1 mg near-infrared fluorescent dye of DiR was used instead of 1 mg monostearin to prepare DiR-labeled ICAM/DEX/NLCs, IgG/DEX/NLCs, ICAM/DEX/ODA-NLCs and IgG/DEX/ODA-NLCs via solvent diffusion method. To establish murine ALI model, the mice were anesthetized and challenged slowly with an intratracheal instillation of LPS (2 mg/kg) [26], followed by stimulation for 6 h. Subsequently, the healthy mice and the ALI model mice were tail-intravenously administrated with 0.2 mL of the DiR-labeled NLCs. After 24 h later, the mice were sacrificed and the lung tissues were excised. A Maestro in vivo imaging system (Cambridge Research & Instrumentation, Inc., Woburn, MA, USA) was used to observe the pulmonary accumulation of the formulated NLCs and semi-quantitatively analyze pulmonary fluorescent signals intensity.

### In vivo pharmacodynamics study

#### Experimental design

All mice were divided into seven groups randomly with six mice per group: control group, ALI model group, dexamethasone treated group, IgG/DEX/ODA-NLCs treated group, ICAM/DEX/ODA-NLCs treated group, IgG/DEX/NLCs treated group and ICAM/DEX/NLCs treated group. The ALI model mice of treatment groups were tail-intravenously administrated with the formulated drugs at a dexamethasone dose of 1.2 mg/kg. While the control and ALI model mice were i.v. administrated with saline. After 12 h and 24 h drug administration, the mice were sacrificed and the bronchoalveolar lavage fluid (BALF) and lungs without bronchoalveolar lavage (BAL) were harvested for next in vivo therapy study.

#### Pulmonary inflammation assessments

The collection of BALF was performed through a tracheal cannula method [26]. In brief, 2 mL of sterile PBS was injected in the lung and slowly withdrawn repeatedly till five times. The harvested BALF samples were centrifuged at 1300 rpm for 10 min followed by storing the cell-free supernatant at − 80 °C. The levels of cytokines including tumor necrosis factor-α (TNF-α) and interleukin-6 (IL-6) in the BALF were determined by ELISA assay (Boster Co., Ltd., Wuhan, China) according to the manufactured protocol. In other to determine the inflammatory cells infiltration, the cell pellet obtained after centrifugation was dispersed in ACK Lysis Buffer to lyse the red blood cells for 2 min on an ice bath, followed by termination with PBS and centrifugation. Afterwards, the collected cell pellet was re-suspended in 0.5 mL PBS. 0.05 mL of the suspensions was used to evaluate the pulmonary total inflammatory cells infiltration through a hemocytometer. 0.45 mL of the suspensions was used to determine the pulmonary neutrophils (PMNs) infiltration through a flow cytometry. Double-labeling experiment of PMNs using FITC rat anti-mouse CD11b (FITC-CD11b, M1/70) and PE rat anti-mouse Ly-6G and Ly-6C (PE-Gr-1, RB6-8C5) antibodies were carried out at 4 °C for 20 min. Then the cells were rinsed using PBS followed by centrifugation, and finally fixed using 2% paraformaldehyde. A flow cytometer was used to detect the ratio of PMNs vs. total cells in the BALF. The PMNs concentration was obtained by the product of total cells concentration and the ratio of PMNs vs. total cells.

#### Pulmonary histopathology examination

The pulmonary histopathology examination was carried out using the murine lung tissues without BAL, performed by hematoxylin and eosin (H&E) stain. The pulmonary tissue samples were immersed in 10% neutral-buffered formalin for 48 h, followed by dissection, dehydration, embedding in paraffin and slicing at a thickness of 5 μm sections successively. Hematoxylin and eosin were used to stain the specimen based on the procedures accurately. A light microscope was finally employed to photograph the pulmonary histopathology characteristics at 200× magnification (Nikon, ECLIPSE Ni).

### Statistics

All data represent the mean ± standard deviation. Statistical significance difference between two groups was assessed with Student’s t-tests. p value < 0.05 was considered as statistically significant.

## Results and discussion

### Physicochemical properties of the NLCs

The DEX-loaded NLCs were prepared by solvent diffusion method firstly. Afterwards, the anti-ICAM-1 antibody or the anti-IgG antibody was conjugated to the nanoparticles surface via a chemical binding between the amino groups of antibody and the amino groups of NH_2_-PEG_2000_-SA mediated by DSC [27]. As the surface charge is one of the dominant factors regulating nanoparticles in vivo behavior, in this study, anionic and cationic DEX-loaded NLCs were prepared as the variables to investigate the effects on ALI. The physicochemical characteristics of the NLCs are summarized in Table 1. The formulated NLCs exhibited similar diameter from about 227.9–249.9 nm, which suggested the modification with 3 wt% octadecylamine has no prominent effect on particle size. The polydispersity index was from about 0.166–0.231, indicating the relatively narrow distribution of the NLCs. The NLCs without ODA modification showed negative surface charges, which were reversed into positive after 3 wt% ODA modification, suggesting the successful preparation of the anionic and cationic NLCs. Besides, good drug encapsulation efficiency from 81.39 to 90.11% and similar drug loading content from 3.28 to 3.62% of the formulated NLCs were also showed in Table 1.

**Table 1:** The characterization of the formulated NLCs

Sample	*d*_*n*_ (nm)	PI	Zeta potential (mV)	EE (%)	DL (%)
ICAM/DEX/NLCs	249.9 ± 21.5	0.231 ± 0.025	− 30.3 ± 0.5	90.11 ± 1.34	3.62 ± 0.05
IgG/DEX/NLCs	229.3 ± 17.2	0.225 ± 0.016	− 28.7 ± 1.1	86.72 ± 0.84	3.49 ± 0.03
ICAM/DEX/ODA-NLCs	235.9 ± 1.8	0.166 ± 0.017	37.4 ± 0.7	82.93 ± 0.94	3.34 ± 0.04
IgG/DEX/ODA-NLCs	227.9 ± 7.4	0.201 ± 0.029	34.2 ± 3.3	81.39 ± 3.23	3.28 ± 0.13

**Fig. 1:**
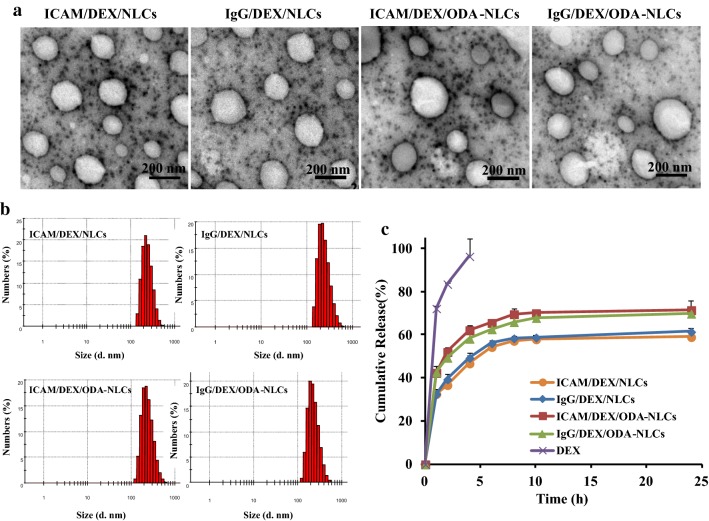
The physicochemical properties of the NLCs. **a** Representative TEM images of ICAM/DEX/NLCs, IgG/DEX/NLCs, ICAM/DEX/ODA-NLCs and IgG/DEX/NLCs (bar = 200 nm). **b** Size distribution of the NLCs analyzed by DLS. **c** The in vitro dexamethasone release from the NLCs at 37 °C in pH 7.4 PBS. The data represent the mean ± SD (n = 3)

Figure 1a, b showed the TEM images and the size distribution of the formulated NLCs obtained from dynamic light scattering (DLS), respectively. All the nanoparticles exhibited the spheroidal morphologies, as well as a similar particle size approximately 200 nm, which was consistent with the diameter estimated by DLS. Figure 1c revealed the formulated DEX-loaded NLCs had a sustained drug release behavior up till to 24 h. Besides, ICAM/DEX/NLCs showed a similar release curve with IgG/DEX/NLCs, suggesting no significant difference of release exists between the NLCs with anti-ICAM-1 antibody conjugation or anti-IgG antibody conjugation.

### The cytotoxicity and cellular uptake study

**Fig. 2:**
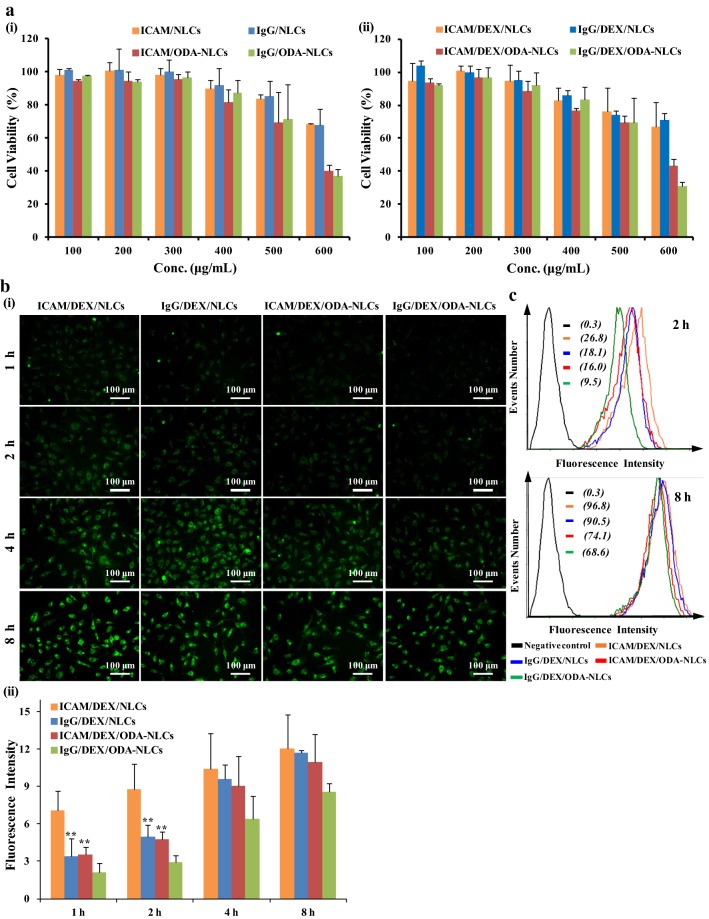
The cytotoxicity and cellular uptake of NLCs against EAhy926 cells. **a** The cytotoxicity of formulated blank NLCs (i) and dexamethasone-loaded NLCs (ii) against activated EAs. **b** Cellular uptake of dexamethasone-loaded NLCs in activated EAs detected by fluorescence microscope (i) (bar = 100 μm) and the semi-quantitative analysis of the images using software ‘‘Image J’’ (ii). **c** Cellular uptake of dexamethasone-loaded NLCs in activated EAs detected by flow cytometer. The data represent the mean ± SD (n = 3)

The EAHy926 is widely used for in vitro endothelial cell research [28]. Given the critical role of pulmonary vascular endothelium as the target for anti-ICAM-1 antibody-modified nanoparticles, EAHy926 was used to study the cellular experiments in this study. EAs were activated using LPS and employed to incubate with blank NLCs and DEX-loaded NLCs for cytotoxicity study by MTT assay. Figure 2a-i showed a dose-dependent cell inhibition effect of the blank NLCs. The 50% cellular growth inhibitions (IC_50_) values against activated EAs of ICAM/NLCs and IgG/NLCs were larger than 600 (μg/mL), indicating the blank carriers without ODA modification had relatively low cytotoxicity on EAs. Nevertheless, the IC_50_ values of ICAM/ODA-NLCs and IgG/ODA-NLCs were smaller than 600 (μg/mL), suggesting that an ODA content of 3 wt% in the NLCs could increase the cytotoxicity of the carriers on EAs. These results might be due to potential stronger damage of plasma-membrane integrity by cationic nanoparticles relative to anionic ones [29]. The cytotoxicity of DEX-loaded NLCs exhibited similar effects tendency (Fig. 2a-ii), which suggested ICAM/DEX/NLCs and IgG/DEX/NLCs without ODA modification displayed as a promising DDS with relatively low cytotoxicity on the endothelial cells.

**Fig. 3:**
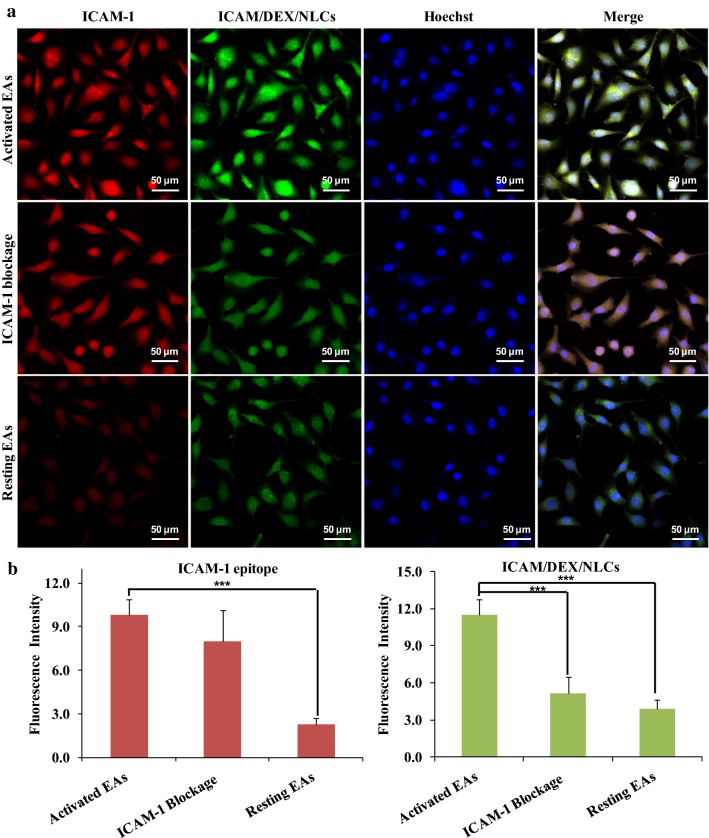
The transport pathway of ICAM/DEX/NLCs in EAhy926 cells. **a** The immunofluorescence analysis of ICAM-1 on cells and the internalized FITC-labeled ICAM/DEX/NLCs in EAs. The nucleus was detected with Hoechst 33342 (bar = 50 μm). **b** The semi-quantitative analysis of the fluorescent images of ICAM-1 epitope and the internalized ICAM/DEX/NLCs by software ‘‘Image J’’. The data represent the mean ± SD (n = 3)

The cellular uptake of FITC-labeled DEX-loaded NLCs in quiescent EAs (Additional file 1: Figure S1) and LPS-activated EAs (Fig. 2b) was carried out by fluorescence microscope. Additional file 1: Figure S1 and Fig. 2b-i exhibited a time-dependent internalization of the NLCs in EAs. Although ICAM/DEX/NLCs showed no significant cellular uptake difference relative to the non-targeted counterparts IgG/DEX/NLCs in quiescent EAs, stronger uptake of ICAM/DEX/NLCs relative to IgG/DEX/NLCs in LPS-activated cells at the same incubated time point was exhibited. The results were more directly observed by the semi-quantitative analysis of the fluorescent pictures using “ImageJ” (Fig. 2b-ii). The results were further confirmed by flow cytometry examination, which showed the cells incubated with ICAM/DEX/NLCs had higher mean fluorescence intensity (MFIs) of 26.8 (2 h) and 96.8 (8 h) than that incubated with IgG/DEX/NLCs after 2 h (18.1) or 8 h (90.5) respectively (Fig. 2c). As ICAM-1 was over-expressed on EAs’ surface after LPS-stimulation (Fig. 3a), the potentially specific recognition and combination of the anti-ICAM-1 antibody to the ICAM-1 on activated EAs was the possible reason facilitating the transendothelial transport of ICAM/DEX/NLCs in activated EAs. Meanwhile, the weak expression of ICAM-1 on quiescent EAs was the potential reason reducing cellular internalization of ICAM/DEX/NLCs, contributing to insignificant difference in cellular uptake between ICAM/DEX/NLCs and IgG/DEX/NLCs in quiescent EAs. Besides, the difference in uptake among the nanoparticles was decreased with incubation time increasing, which possibly was due to the condition that uptake by EAs reached saturation around 8 h.

Noteworthily, the anionic ICAM/DEX/NLCs showed lower uptake efficiency than the cationic ICAM/DEX/ODA-NLCs in quiescent EAs (Additional file 1: Figures S1, S2), whereas the uptake results were reversed in activated EAs under inflammatory pathological condition (Fig. 2b). The flow cytometry examination also confirmed higher uptake efficiency of anionic ICAM/DEX/NLCs than cationic ICAM/DEX/ODA-NLCs in activated EAs (Fig. 2c). The results suggested that although cationic ICAM/DEX/ODA-NLCs owned higher uptake than anionic ICAM/DEX/NLCs in quiescent EAs due to the potential electrostatic interaction with negatively charged cell membrane of the former, whereas anionic ICAM/DEX/NLCs may be more favorable for cellular uptake mediated by CAM-mediated endocytosis in activated EAs with over-expressed ICAM-1 epitope. Similar higher cellular internalization of anionic nanoparticles than the cationic ones has also been revealed in previous studies [30–33]. Overall, ICAM/DEX/NLCs showed promising cellular internalization ability in activated EAs, which is of great significance to increase DEX accumulation in inflammatory endothelium and enhance endothelial intervention.

### Transport pathway of ICAM/DEX/NLCs

The transport pathway of ICAM/DEX/NLCs in EAs was further investigated after 2 h incubation using a confocal microscopy. Figure 3a exhibits the internalization results of the FITC-labeled ICAM/DEX/NLCs in LPS-activated EAs, activated EAs with ICAM-1 epitopes blockage and resting EAs, respectively. The red fluorescent signals reflected the expression level of ICAM-1 epitopes on the EAs, while the green signals and blue signals signified the internalized ICAM/DEX/NLCs and the cell nucleus with Hochest 33342 staining. As Fig. 3a showed, the activated EAs showed significantly higher red fluorescence signals relative to the resting EAs, suggesting the ICAM-1 was over-expressed on the cells surface after LPS stimulation. After 2 h incubation, significantly more ICAM/DEX/NLCs were internalized in activated EAs than that in resting EAs. Meanwhile, the internalization of ICAM/DEX/NLCs in activated EAs was reduced after ICAM-1 blockage. The results were more directly observed by the semi-quantitative analysis of the fluorescent pictures using “ImageJ” (Fig. 3b), and were also further demonstrated by flow cytometry quantitatively (Additional file 1: Figure S3). Besides, Fig. 2b also showed that ICAM/DEX/NLCs exhibited higher cellular uptake than IgG/DEX/NLCs in activated EAs, especially after a short incubation. These results directly suggested that the internalization of ICAM/DEX/NLCs was associated with ICAM-1 epitopes, indicating the widely accepted CAM-mediated endocytosis [34] was the potential internalization mechanism of anti-ICAM-1 antibody-conjugated nanoparticles in this study. Similar results were also demonstrated in our previous study, suggesting the anti-ICAM-1 antibody-conjugated nanoparticles were internalized by LPS-activated EAs through CAM-mediated endocytosis [35].

### Pulmonary-distribution of NLCs

**Fig. 4:**
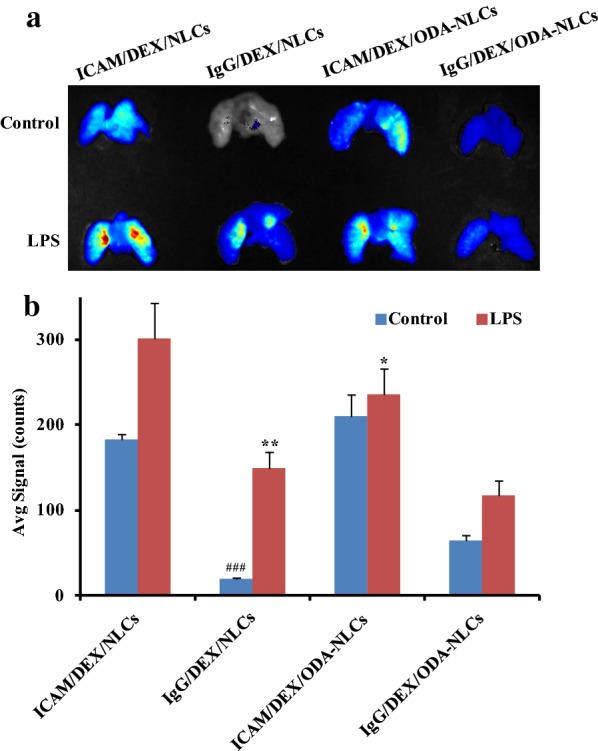
In vivo pulmonary distribution. **a** The pulmonary distribution of ICAM/DEX/NLCs, IgG/DEX/NLCs, ICAM/DEX/ODA-NLCs and IgG/DEX/NLCs in healthy mice and LPS-induced ALI mice after 24 h drug administration. **b** The semi-quantitative fluorescence intensity in the lungs, expressed as the average fluorescence intensity (avg signal/counts). ^###^p < 0.01 indicated the pulmonary fluorescence intensity of IgG/DEX/NLCs treated group compared with that of ICAM/DEX/NLCs treated group in healthy mice. *p < 0.05, **p < 0.01 indicated the pulmonary fluorescence intensity of corresponding groups compared with that of ICAM/DEX/NLCs treated group in ALI mice

Pulmonary distribution of the formulated NLCs was determined in healthy mice and LPS-challenged ALI model mice. As shown in Fig. 4, both of the healthy mice and the ALI model mice administrated with ICAM/DEX/NLCs showed stronger pulmonary fluorescence signals than that of the mice given non-targeted IgG/DEX/NLCs. The pulmonary distribution superiority of ICAM/DEX/NLCs relative to IgG/DEX/NLCs was potentially due to multivalent interactions of anti-ICAM-1 antibody on ICAM/DEX/NLCs with the ICAM-1 receptors on pulmonary endothelium, which may contribute to the binding of NLCs on endothelium surface sites followed by endocytosis. Meanwhile, it was also observed that although anionic ICAM/DEX/NLCs exhibited no superiority of pulmonary distribution relative to cationic ICAM/DEX/ODA-NLCs in healthy mice, the anionic ones showed significant higher pulmonary distribution than the cationic ones in ALI model mice (p < 0.05). It is relatively widely recognized that the cationic particles potentially could be internalized by cells easier than the anionic ones due to the electrostatic interaction with negatively charged cell membrane of the former. However, in vivo pulmonary distribution results suggested anionic ICAM/DEX/NLCs exhibited higher cellular internalization in pulmonary endothelium under inflammatory pathological states than cationic ICAM/DEX/ODA-NLCs, which was similar with the in vitro cellular uptake results. The phenomenon potentially could be still explained by that the anionic ICAM/DEX/NLCs favored the uptake in activated endothelium with over-expressed ICAM-1 mediated by CAM-mediated endocytosis. Collectively, the pulmonary distribution advantage of anionic ICAM/DEX/NLCs in ALI model mice mediated by endothelial ICAM-1-targeting was suggested here.

### In vivo pharmacodynamics assessments

The in vivo therapeutic efficacy of ICAM/DEX/NLCs, IgG/DEX/NLCs, ICAM/DEX/ODA-NLCs and IgG/DEX/ODA-NLCs was investigated using a widely studied LPS-induced murine model of ALI [26]. LPS, a main component of gram-negative bacterial cell wall involved in sepsis-mediated ALI, could act on the Toll-like receptor 4 to exacerbate inflammatory cascades in lung, pulmonary inflammatory cells infiltration and hemorrhage, etc. Thus the related inflammatory mediators in lung were measured after 12 h and 24 h drug administration for pharmacodynamics assessments.

**Fig. 5:**
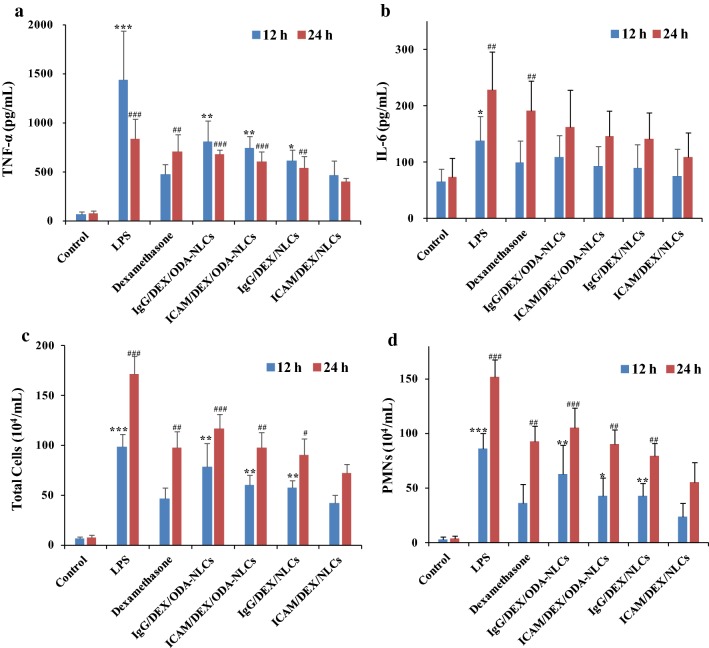
In vivo anti-inflammatory evaluation. **a** TNF-α level. **b** IL-6 level. **c** Total cells counts. **d** Neutrophils (PMNs) counts. *p < 0.05, **p < 0.01 and ***p < 0.001 indicated the indices of corresponding groups compared with ICAM/DEX/NLCs treated groups after 12 h drug administration. ^#^p < 0.05, ^##^p < 0.01 and ^###^p < 0.001 indicated the indices of corresponding groups compared with ICAM/DEX/NLCs treated groups after 24 h drug administration. The data represent the mean ± SD (n = 6)

The over-expressed pro-inflammatory cytokines such as TNF-α and IL-6 could aggravate ALI. The attenuation of pro-inflammatory cytokines is a significant method for ALI therapy. Figure 5a, b showed the drugs effects on TNF-α and IL-6 levels in BALF of the ALI mice respectively. Upon the LPS challenge via air-way, a pronouncedly increased TNF-α level relative to the control group was observed (~ 1441.6 pg/mL at 12 h, ~ 834.2 pg/mL at 24 h, Fig. 5a). Different down-regulation of the TNF-α after the formulated drugs administration was revealed. Thereinto, ICAM/DEX/NLCs exhibited a significant inhibition of TNF-α relative to the other drugs after 24 h administration. Relative to the other preparations in this study, the potentially increased pulmonary delivery of dexamethasone by ICAM/DEX/NLCs due to their pulmonary distribution superiority in the ALI model mice (Fig. 4) and positive cellular uptake characteristic (Fig. 2b) were the possible reasons contributed to the most effective attenuation of TNF-α. Meanwhile, it is worth to mention that both ICAM/DEX/NLCs and free dexamethasone significantly reduced pulmonary inflammation of ALI model after 12 h drug administration, while statistical significance of efficacy between free dexamethasone and ICAM/DEX/NLCs treatment groups was not reached. This may be due to the prolonged drug release in nanoparticles. The sustained release may also contribute to the reduction of drugs leakage in the bloodstream, decreasing system side effects and increasing the dexamethasone content delivered into the pathological pulmonary endothelium. The tendency of the inhibition for IL-6 in BALF by the formulated drugs was similar with their effects on TNF-α (Fig. 5b). Collectively, the data suggested the therapeutic effects of ICAM/DEX/NLCs on LPS-induced ALI in mice by effectively down-regulating the pro-inflammatory cytokines.

The infiltration of inflammatory cells in lung resulting from high pulmonary capillary permeability is another pathological basis of ALI. Thereinto the infiltrated PMNs after activation could produce serine proteases, reactive oxygen species, cytokines and so on to further damage the lung. Therefore, the therapy of ALI by the drugs can also be evaluated via their inhibition effects on pulmonary inflammation leakage. Figure 5c, d showed the attenuation effects of total inflammatory cells and PMNs infiltration in lung by the drugs, respectively. The PMNs were detected using FITC-labeled anti-CD11b antibody and PE-labeled anti-Gr-1 antibody to specifically identify their typical epitopes of CD11b and Gr-1 (FITC-CD11b+, PE-Gr-1+) [36]. It was found that the total inflammatory cell number in BALF was dramatically up-regulated with severe PMNs infiltration after LPS-stimulation. Similar to the results of pro-inflammatory cytokines assessments, ICAM/DEX/NLCs significantly reduced the LPS-induced pulmonary migration of total inflammatory cells and PMNs after 24 h i.v. administration compared with the other formulations. The results indicated the anti-inflammatory activities of ICAM/DEX/NLCs on the LPS-induced ALI mice by attenuating inflammatory cells infiltration.

**Fig. 6:**
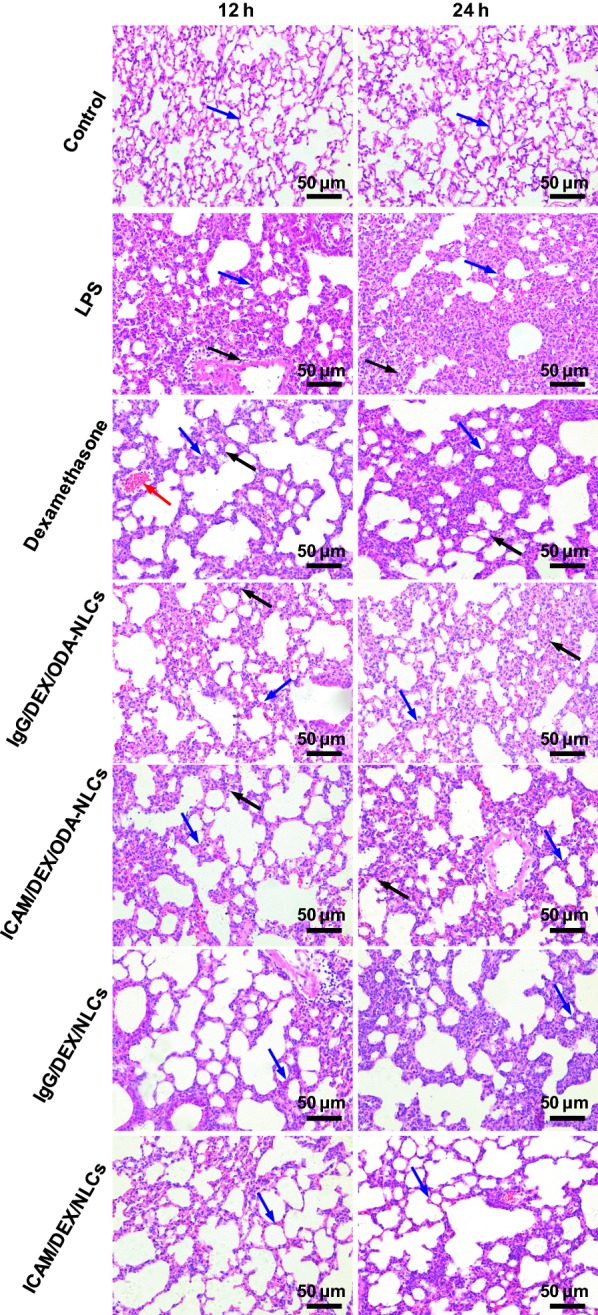
The pulmonary histopathological examination by H&E stain. The black arrows depict the neutrophils infiltration. The red arrows indicate the alveolar wall hyperemia. The blue arrows indicate the alveolar wall (bar = 50 μm)

Additionally, H&E stain was carried out to evaluate the efficacy of the NLCs by observing pulmonary histomorphology. Severe alveolar walls thickening, congestion and dramatical inflammatory cells infiltration was observed in the pulmonary sections of ALI model mice (Fig. 6). The remarkable suppression effects on inflammatory cells infiltration, alveolar wall thickening and so on by the formulated drugs were revealed. Thereinto ICAM/DEX/NLCs showed the significant improvements of pathological lung tissues relative to the drugs after 12 h and 24 h administration, which was consistent with the quantitative detection of pro-inflammatory cytokines and inflammatory cells infiltration improvement in BALF.

## Conclusions

In our work, the pulmonary vascular endothelium-targeted NLCs mediated by anti-ICAM-1 antibody with negative surface charge or positive surface charge were developed to delivery DEX to lung and explore an alternative pathway for ALI therapy. Our study demonstrated that the anionic ICAM/DEX/NLCs could encapsulate dexamethasone effectively, and display low cytotoxicity, significant enhancement cellular uptake in LPS-activated EAs, as well as the advantage of pulmonary distribution. In vivo pharmacodynamics study demonstrated that the anionic ICAM/DEX/NLCs showed significant anti-inflammation efficacy on attenuating TNF-α, IL-6 and inflammatory cells infiltration in BALF. The positive pulmonary tissue structure improvement by ICAM/DEX/NLCs was further confirmed through H&E stain. The results suggested that, ICAM/DEX/NLCs may represent a potential lung-targeting candidate favoring ALI therapy in clinic.

## Additional file

**Additional file 1: Figure S1.** Cellular uptake of dexamethasone-loaded NLCs in quiescent EAs detected by fluorescence microscope (bar = 100 μm). **Figure S2.** Cellular uptake of dexamethasone-loaded NLCs in quiescent EAs detected by flow cytometry. **Figure S3.** Transport pathway study of ICAM/DEX/NLCs in EAhy926 cells analyzed by Flow cytometry.
