# Supplementary material for: Anti-ICAM-1 antibody-modified nanostructured lipid carriers: a pulmonary vascular endothelium-targeted device for acute lung injury therapy
Source: J Nanobiotechnology. 2018 Dec 29;16:105. doi: 10.1186/s12951-018-0431-5 (PMC6311082; doi:10.1186/s12951-018-0431-5)
Supplement: Supplementary file 1 — Additional file 1: Figure S1. Cellular uptake of dexamethasone-loaded NLCs in quiescent EAs detected by fluorescence microscope (bar = 100 μm). Figure S2. Cellular uptake of dexamethasone-loaded NLCs in quiescent EAs detected by flow cytometry. Figure S3. Transport pathway study of ICAM/DEX/NLCs in EAhy926 cells analyzed by Flow cytometry. [file 12951_2018_431_MOESM1_ESM.docx]

**Additional file 1**

**Figure S1**. Cellular uptake of dexamethasone-loaded NLCs in quiescent EAs detected by fluorescence microscope (bar= 100 μm).

**Figure S2**. Cellular uptake of dexamethasone-loaded NLCs in quiescent EAs detected by flow cytometry. The mean fluorescence intensity of each group has been normalized to the mean fluorescence intensity value of the cells incubated with ICAM/DEX/NLCs for 2 h. ***p<0.001 indicated the mean fluorescence intensity (MFIs) of the cells incubated with ICAM/DEX/ODA-NLCs compared with that of the cells incubated with ICAM/DEX/NLCs. The data represented the mean± SD (n=3).

**Figure S3**. Transport pathway study of ICAM/DEX/NLCs in EAhy926 cells analyzed by Flow cytometry. The mean fluorescence intensity of each group has been normalized to the mean fluorescence intensity value of the activated EAs incubated with ICAM/DEX/NLCs. **p<0.01 indicated the mean fluorescence intensity (MFIs) of the corresponding groups compared with ICAM/DEX/NLCs treated group. The data represented the mean± SD (n=3).
